# The role of capsule stiffness on cellular processing†
†Electronic supplementary information (ESI) is available. See DOI: 10.1039/c5sc00416k



**DOI:** 10.1039/c5sc00416k

**Published:** 2015-04-29

**Authors:** Huanli Sun, Edgar H. H. Wong, Yan Yan, Jiwei Cui, Qiong Dai, Junling Guo, Greg G. Qiao, Frank Caruso

**Affiliations:** a ARC Centre of Excellence in Convergent Bio-Nano Science and Technology , and the Department of Chemical and Biomolecular Engineering , The University of Melbourne , Parkville , Victoria 3010 , Australia . Email: fcaruso@unimelb.edu.au; b Department of Chemical and Biomolecular Engineering , The University of Melbourne , Parkville , Victoria 3010 , Australia . Email: gregghq@unimelb.edu.au

## Abstract

Particle stiffness is emerging as an important parameter in determining the cell uptake dynamics of particles. Understanding the effects of capsule stiffness on their biological behavior is essential for the development of polymer capsules as therapeutic carriers. Herein, we report the preparation of polysaccharide capsules *via* atom transfer radical polymerization-mediated continuous assembly of polymers (CAP_ATRP_) on silica templates using methacrylated hyaluronic acid (HA) as the macrocrosslinker. This approach affords HA capsules with controllable wall thickness and tunable stiffness. The influence of capsule stiffness on cellular interaction and intracellular distribution is systematically investigated using flow cytometry, imaging flow cytometry, and deconvolution microscopy. The softest HA capsules with a stiffness (*γ*) of 7.5 mN m^–1^ possess higher cell surface binding and cellular association when compared to stiffer capsules with *γ* of 17.6–28.9 mN m^–1^. Furthermore, the uptake of HA capsules is a stiffness-dependent process, with slower and less cellular internalization observed with increasing capsule stiffness. Nevertheless, regardless of the stiffness, all internalized capsules are deformed and located in the lysosomes. These findings offer insights into the influence of capsule stiffness on cellular interaction as well as intracellular fate, providing information for the design of rational polymer capsules for biomedical applications.

## Abstract 2

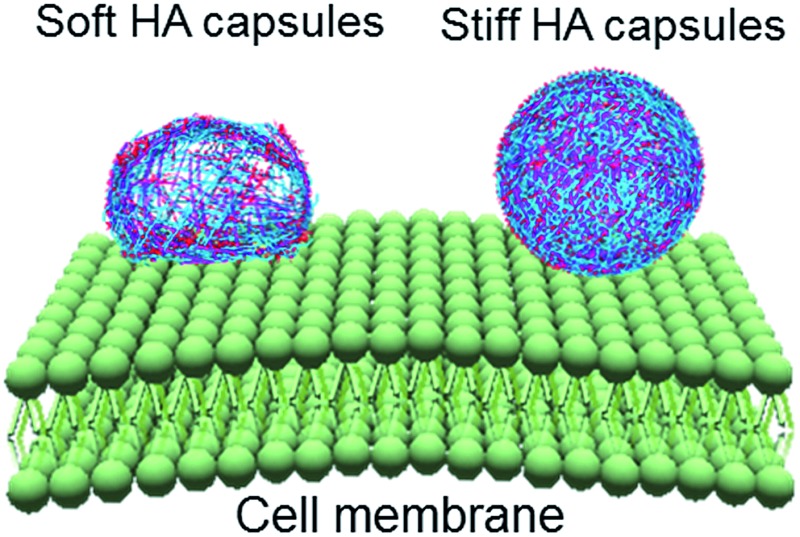
A systematic and quantitative study on the role of capsule stiffness in cellular processing was performed using hyaluronic acid capsules with tunable stiffness constructed *via* continuous assembly of polymers.

## Introduction

In the past decades, polymer particles have emerged as a promising therapeutic platform for the treatment of diseases due to the ability to tune their composition and properties.^1,2^ It is well-known that the physicochemical properties of particles such as size, shape, surface chemistry, and rigidity have significant effects on their biological behavior.^3–5^ Therefore, establishing correlations between the physicochemical properties of particles and their biological performance is important toward the development of the next generation of polymer particles for efficient therapeutic delivery.

To date, many studies have been performed to determine the influence of particle size, surface chemistry, and shape on biological interactions.^6,7^ Studies have also been conducted to shed light on the importance of particle stiffness on biological processes.^8–12^ Particle stiffness has been shown to influence the particle behavior including *in vivo* circulation and *in vitro* cellular interactions.^13–18^ For example, 2-hydroxyethyl acrylate (HEA)-based red blood cell mimic (RBCM) microparticles with a Young's modulus (*E*_Y_) of 7.8 kPa were demonstrated to pass through narrow microchannels *via* reversible elastic deformation, whereas the stiffer particles (63.9 kPa) stuck at the entrance.^13^ Accordingly, softer RBCM microparticles and nanogels exhibit longer *in vivo* circulation time and lower splenic accumulation compared to their stiffer analogues with higher crosslinking degree.^13,14^ In addition, studies on particle stiffness-related cell interaction have shown that particle stiffness affects cell interaction efficiency, although with varied results.^15–18^ For instance, hydrogel nanoparticles (*ca.* 150 nm in diameter) composed of *N*,*N*-diethyl acrylamide and 2-hydroxyethyl methacrylate (HEMA) with intermediate stiffness (35–136 kPa) revealed a faster and higher uptake into macrophage RAW 264.7 cells.^16^ In a separate study, softer HEMA hydrogel particles (*ca.* 17–30 kPa) with a diameter of 800–1000 nm were internalized into HepG2 cells more rapidly and efficiently compared to the stiffer particles (*ca.* 76–156 kPa).^17^ Conversely, softer poly(l-glutamic acid)-CpG particles with a diameter of 800 nm exhibited lower cell association toward plasmacytoid dendritic cells.^18^ In general, there is a lack of consensus on how particle stiffness influences cellular interactions. One of the reasons could be the crosslinking strategy used to control the particle stiffness, which can alter the particle composition and surface chemistry.^5^ Therefore, to decouple the combined effects, it is important to develop new approaches for controlling particle stiffness without altering other particle parameters, thus providing a better understanding on the importance of stiffness in biological interactions.

Polymer capsules,^19,20^ a unique category of polymer particles with a versatile polymeric shell and a hollow cavity, provide a convenient platform to control particle stiffness *via* manipulating the capsule shells. Several approaches have been reported to tune the capsule stiffness, which include incorporating metal nanoparticles,^21,22^ thermal shrinking,^23^ and tailoring shell thickness.^12,24,25^ For instance, by doping with gold nanoparticles and/or thermal shrinking, the stiffness of poly(diallyldimethylammonium chloride)/poly(styrenesulfonate) (PDADMAC/PSS) capsules increased, which consequently enhanced the resistance of capsule to deformation during cell internalization. Capsules doped with 20 nm gold nanoparticles and shrunk to half of their original size showed higher mechanical stability with 80% of capsules remaining intact after internalization.^22^ By tuning the shell thickness from 150 to 800 nm, poly(allylamine hydrochloride)/PSS (PAH/PSS) capsules with different stiffness were obtained, while these capsules all deformed during the uptake process by HeLa cells.^24^ Similarly, by altering the number of bilayers from 2 to 16 in layer-by-layer (LbL) assembly, PAH/PSS and dextran sulfate sodium salt/poly-l-arginine hydrochloride (DextS/PLArg) capsules with diverse stiffness were obtained. Subsequent cell uptake studies revealed that capsule stiffness influenced their uptake and endosomal trafficking time, which prolonged with increasing capsule stiffness (<5 N m^–1^).^12^ In a separate report, protein capsules with a wall thickness of 6 nm showed higher association to HeLa cells compared to those with thicker shells (8–14 nm).^26^ Despite these investigations, a quantitative and systematic stiffness-related biological study of polymer capsules focusing on cell membrane binding, cellular association and internalization remains to be performed.

Recently, we presented a new thin-film fabrication approach, termed continuous assembly of polymers (CAP), to yield polymer capsules *via* one-step polymerization of prefunctionalized polymers (referred to as macrocrosslinkers) from initiator-immobilized surfaces.^27–29^ The CAP approach is amenable to a wide range of macrocrosslinkers consisting of pendent vinylic groups and can effectively tune the film composition and thickness.^30,31^ Previously, we have demonstrated a near-linear growth of film thickness with the increase in CAP step number for PHEMA, PHEA and poly(methyl methacrylate) (PMMA) polymers.^27–29^ Utilizing the CAP approach, polymer capsules with controllable film thickness, and consequently tunable stiffness can be readily made and employed in stiffness-related biological studies.

**Scheme 1:**
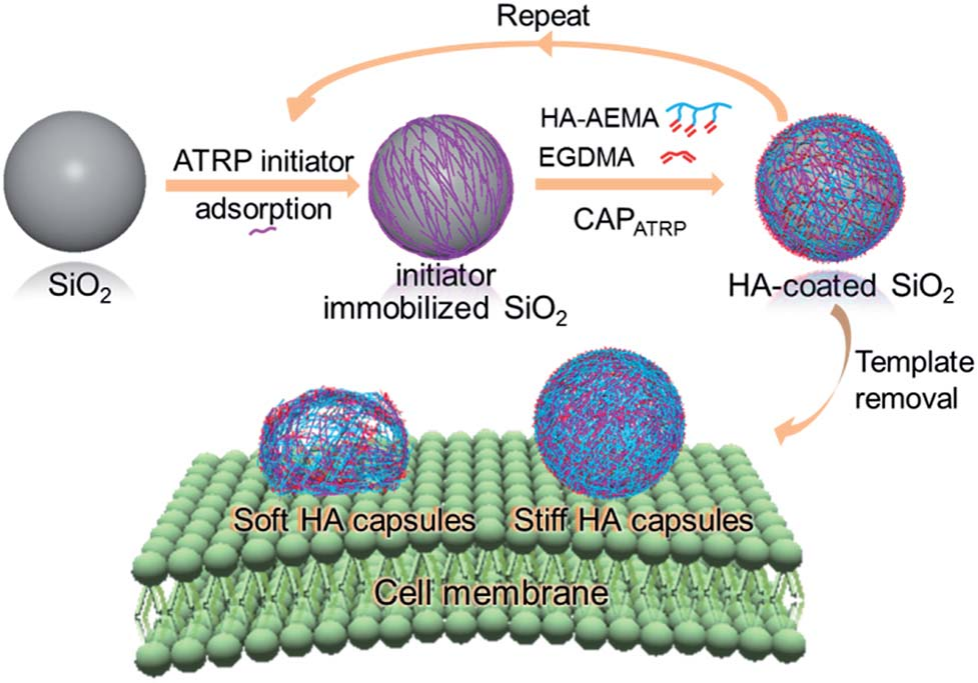
Illustration of HA capsule formation and their cell surface binding behavior. Capsules are prepared *via* CAP_ATRP_ using SiO_2_ particles as templates, which includes ATRP initiator adsorption, ATRP-promoted HA film growth, and template removal.

Herein, we report a systematic and quantitative study on the role of polymer capsule stiffness on cellular interactions. A natural polysaccharide, hyaluronic acid (HA), was chosen to construct the capsules due to its unique properties, such as excellent biocompatibility, non-immunogenicity, and non-inflammation.^32,33^ Specifically, capsules with varying wall thicknesses and stiffness are prepared by atom transfer radical polymerization-mediated continuous assembly of polymers (CAP_ATRP_) of methacrylate-functionalized HA (HA-AEMA) on sacrificial silica (SiO_2_) particles (Scheme 1). The capsule wall thickness and stiffness are determined using atomic force microscopy (AFM). The cytotoxicity of HA capsules toward the HeLa cell line is evaluated *via* XTT assays. Additionally, the cellular interaction and intracellular fate of HA capsules with different stiffness in HeLa cells are investigated *via* flow cytometry, imaging flow cytometry, and deconvolution microscopy. Taken together, this study demonstrates the application of the CAP approach to construct polymer capsules with controllable wall thickness as well as tunable stiffness, and presents insights into the cellular interactions influenced by the stiffness of polymer capsules.

## Results and discussion

The HA-AEMA macrocrosslinker was synthesized from commercially available HA sodium salt (*M*_w_ 47 kDa), wherein the carboxylic groups were partially converted into polymerizable methacrylate groups (12 mol% with respect to the carboxylic acid groups, as determined by ^1^H NMR analysis) *via* reaction of HA with 2-aminoethyl methacrylate (AEMA) in the presence of 4-(4,6-dimethoxy-1,3,5-triazin-2-yl)-4-methylmorpholinium chloride (DMTMM) (Scheme S1 and Fig. S1, ESI†). The ATRP macroinitiator P(METAOTs-*co*-BIEM) (*M*_w_ 7.7 kDa) was obtained by conventional free radical copolymerization of methacrylate monomers containing quaternary ammonium (METAOTs) and ATRP initiator (BIEM) functionalities, where the percentage of BIEM is *ca.* 48 mol% based on ^1^H NMR analysis (Scheme S2, ESI†).

**Fig. 1:**
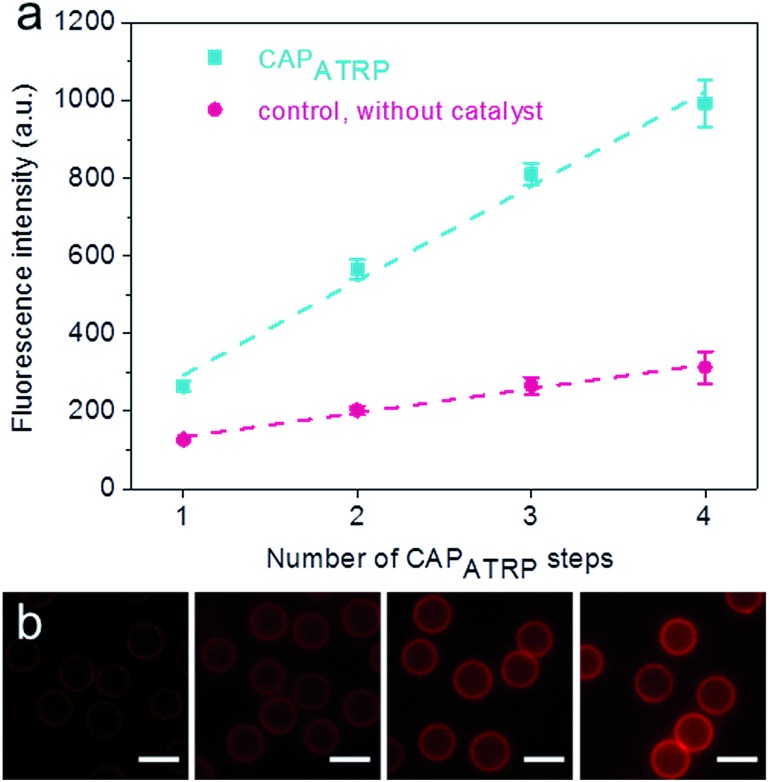
Growth of HA films on SiO_2_ particles. (a) Fluorescence intensity evolution of HA film growth on SiO_2_ particles as a function of CAP_ATRP_ step number, as followed by flow cytometry. (b) Fluorescence microscopy images of HA films deposited on SiO_2_ particles as a function of the number of CAP_ATRP_ steps (from left to right, *n* = 1–4). Scale bars are 3 μm.

SiO_2_ particles (2.4 μm in diameter) were modified with the ATRP macroinitiator P(METAOTs-*co*-BIEM) by electrostatic interactions prior to CAP_ATRP_ in the presence of the HA-AEMA macrocrosslinker (Scheme 1). Reinitiation-film growth steps were performed in the same way to afford multilayered HA films with different thicknesses, where the ATRP macroinitiator was deposited on the preformed HA layers, followed by CAP_ATRP_. To finely monitor the HA film growth, flow cytometry was utilized to detect the fluorescence intensity of SiO_2_ particles using an Alexa Fluor 633 (AF633) labeled HA-AEMA macrocrosslinker. Notably, the CAP_ATRP_ reaction proceeded very quickly at room temperature, as indicated by the rapid increase of the fluorescence intensity of SiO_2_ particles, which reached a plateau after 0.5 h reaction (Fig. S2, ESI†). Moreover, the fluorescence intensity data displayed a near-linear and continuous growth of the HA film with increasing number of CAP_ATRP_ steps. The fluorescence intensity of SiO_2_ particles increased from 96 ± 19 au (*n* = 0) with an increment of *ca.* 243 au per CAP_ATRP_ step to 993 ± 62 au after four CAP_ATRP_ steps (*n* = 4) (Fig. 1a). In comparison, the control experiments performed without any polymerization catalyst (*i.e.*, copper(ii) bromide, sodium ascorbate and *N*,*N*,*N*′,*N*′,*N*′′-pentamethyldiethylenetriamine) revealed only a slight increase in fluorescence intensity to *ca.* 313 ± 41 au (*n* = 4) (Fig. 1a). Fluorescence microscopy images of HA-coated particles after each CAP_ATRP_ step also illustrated HA film generation on SiO_2_ particles, as indicated by the increase in the fluorescence of the particles (Fig. 1b).

**Fig. 2:**
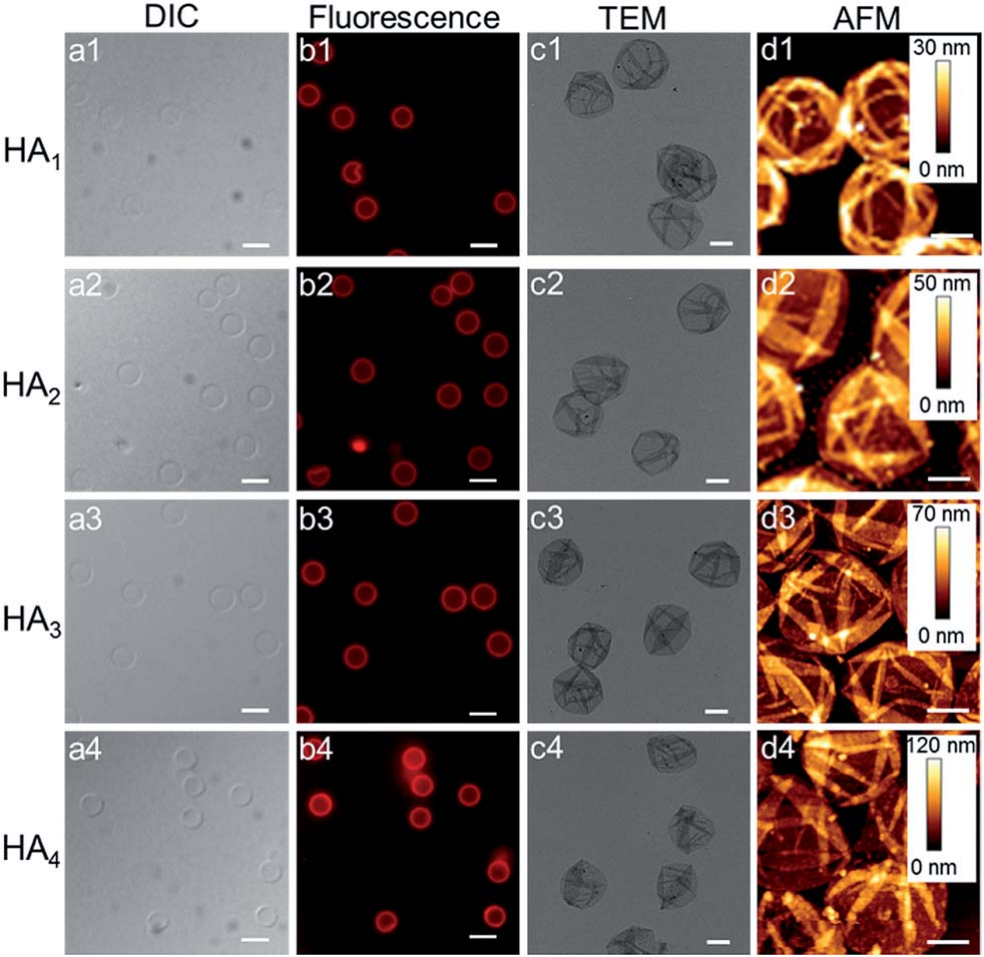
DIC microscopy (a1–a4), fluorescence microscopy (b1–b4), TEM (c1–c4) and AFM (d1–d4) images of HA capsules (HA_1_, HA_2_, HA_3_ and HA_4_) after 1 to 4 CAP_ATRP_ steps, respectively. SiO_2_ particles (2.4 μm in diameter) were used as templates. Scale bars are 3 μm for DIC and fluorescence microscopy images, and 1 μm for TEM and AFM images.

Upon exposure of HA-coated particles after each CAP_ATRP_ step (*n* = 1–4) in hydrofluoric acid (HF) to remove the underlying SiO_2_ templates, homogenous and well-dispersed capsules with negligible shrinkage were obtained, as shown by the corresponding differential interference contrast (DIC, a1–a4) and fluorescence microscopy (b1–b4) images in Fig. 2. Transmission electronic microscopy (TEM) (Fig. 2c1–c4) and AFM images (Fig. 2d1–d4) of different layered HA capsules in the air dried state all showed collapsed structures with folds and creases, typical features that have been observed for air-dried capsules assembled by LbL^34,35^ and metal-phenol coordination^36,37^ techniques.

**Fig. 3:**
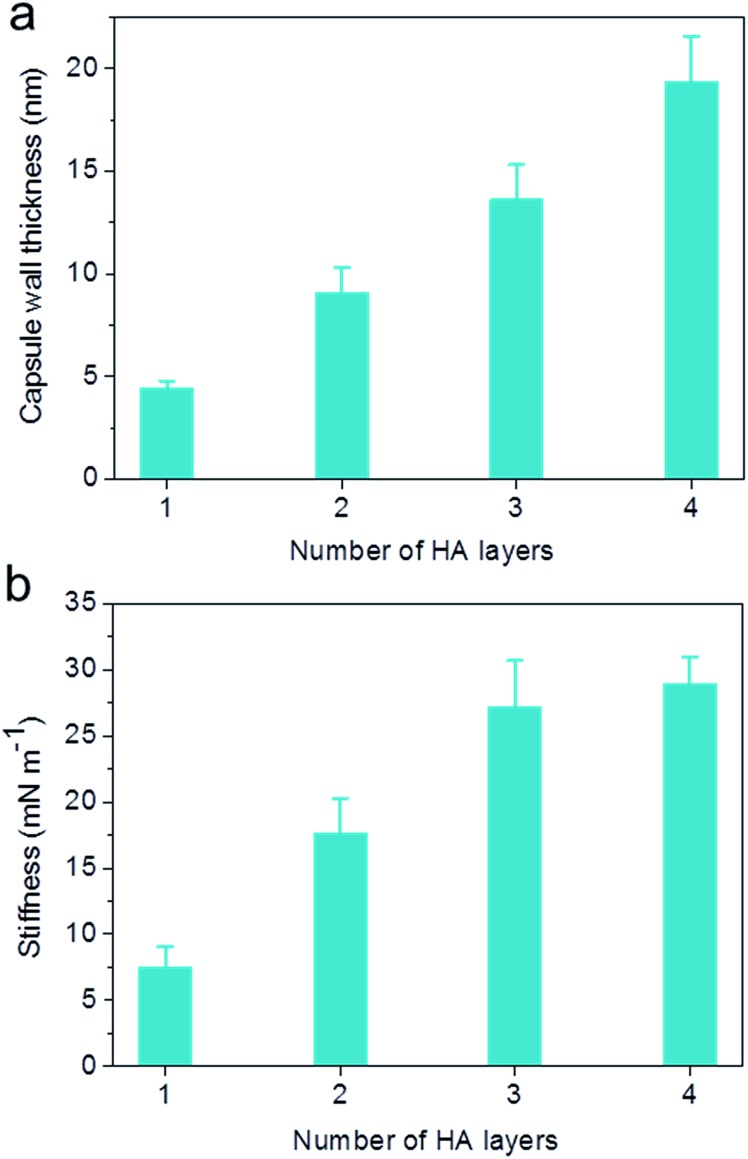
Evolution of (a) capsule wall thickness and (b) stiffness (*γ*) as a function of the number of HA layers, as determined by AFM analysis.

The capsule wall thickness (Fig. 3a), determined by AFM height analysis, increased linearly with increasing CAP_ATRP_ step, which is in line with the fluorescence intensity data and fluorescence microscopy images (Fig. 1a and b). The wall thickness of HA_1_ capsules (4.4 ± 0.4 nm) (formed after one CAP_ATRP_ step; note HA_*n*_ where *n* denotes the number of CAP_ATRP_ steps performed) increased by an average thickness increment of *ca.* 4.8 nm per HA layer until a final wall thickness of *ca.* 19.4 ± 2.2 nm was attained after the fourth CAP_ATRP_ step. The uniform film growth on SiO_2_ particles is likely due to the consistent macroinitiator adsorption and efficient ATRP reaction of the HA-AEMA macrocrosslinker. The mechanical properties of different layered HA capsules were explored using AFM force measurements. *γ* of HA capsules increased linearly with an increase in HA layer up to three layers, for which *γ* of 7.5, 17.6 and 27.2 mN m^–1^ was observed for HA_1_, HA_2_ and HA_3_ capsules, respectively (Fig. 3b). However, *γ* reached a plateau (∼28.9 mN m^–1^) after the fourth HA layer built up (Fig. 3b). The leveling off of mechanical properties (*γ* and *E*_Y_) with an increase in shell thickness has also been observed for LbL capsules.^12,38^ *γ* and *E*_Y_ are intrinsic material properties, which typically has a bulk value determined by the compositional materials. Upon reaching the critical value, *γ* and *E*_Y_ will be thickness independent.^12,39^

The stability of different layered HA capsules was investigated in DMEM medium with the addition of 10% fetal bovine serum (FBS) using flow cytometry. All HA capsules showed good stability in the cell culture medium, for which 89–105% of capsules remained at the end of the assay (Fig. S3, ESI†). The cytotoxicity of different layered HA capsules was evaluated in HeLa cells *via* XTT assays. The results revealed that all HA capsules of different wall thicknesses were non-toxic to HeLa cells (cell viability ranged from 92 to 112%) up to a tested dose of 200 capsules per cell (Fig. S4, ESI†). The following cellular interactions were investigated within the dose range that showed negligible impact on cell viability.

**Fig. 4:**
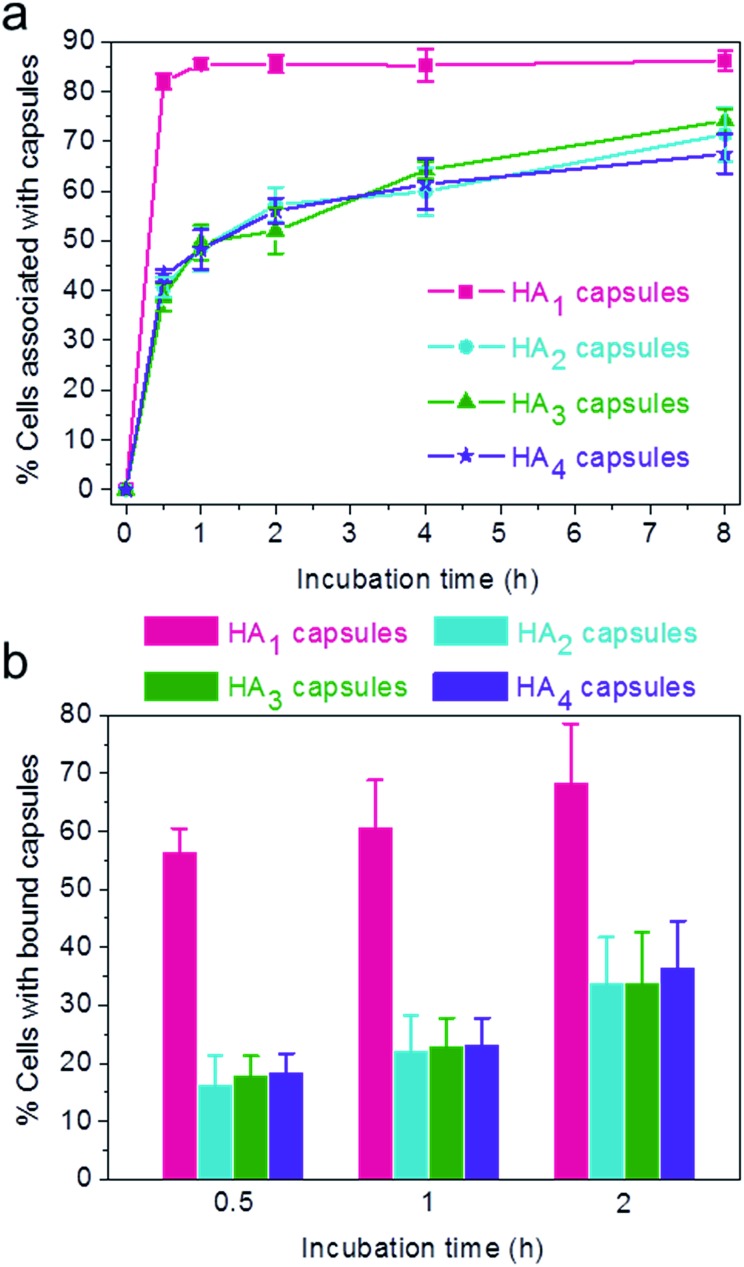
(a) Cellular association (37 °C) and (b) cell surface binding (4 °C) of AF633-labeled HA capsules composed of different HA layers with HeLa cells. Percentage of cells associated or bound with capsules was quantified *via* flow cytometry.

Previous reports have demonstrated that particle stiffness can affect the cell internalization pathway and cellular processing. Hence, we investigated the effect of capsule stiffness on the cellular interaction behavior of HA capsules *via* flow cytometry. Firstly, the cell association study was performed at 37 °C by incubation of HeLa cells with AF633-labeled HA capsules of different stiffness (HA_1_, HA_2_, HA_3_ and HA_4_) at a capsule to cell ratio of 100 : 1 for varying time intervals (0.5, 1, 2, 4 and 8 h). Importantly, the efficiency of cellular association with HA_1_ capsules was significantly higher compared to other multilayered HA capsules (HA_2_, HA_3_ and HA_4_) for all time intervals studied (Fig. 4a). The preferential cellular association with the softest HA_1_ capsules was more significant after 0.5 h incubation, for which 82% (*vs*. 39–43%) of cells associated with capsules.

**Fig. 5:**
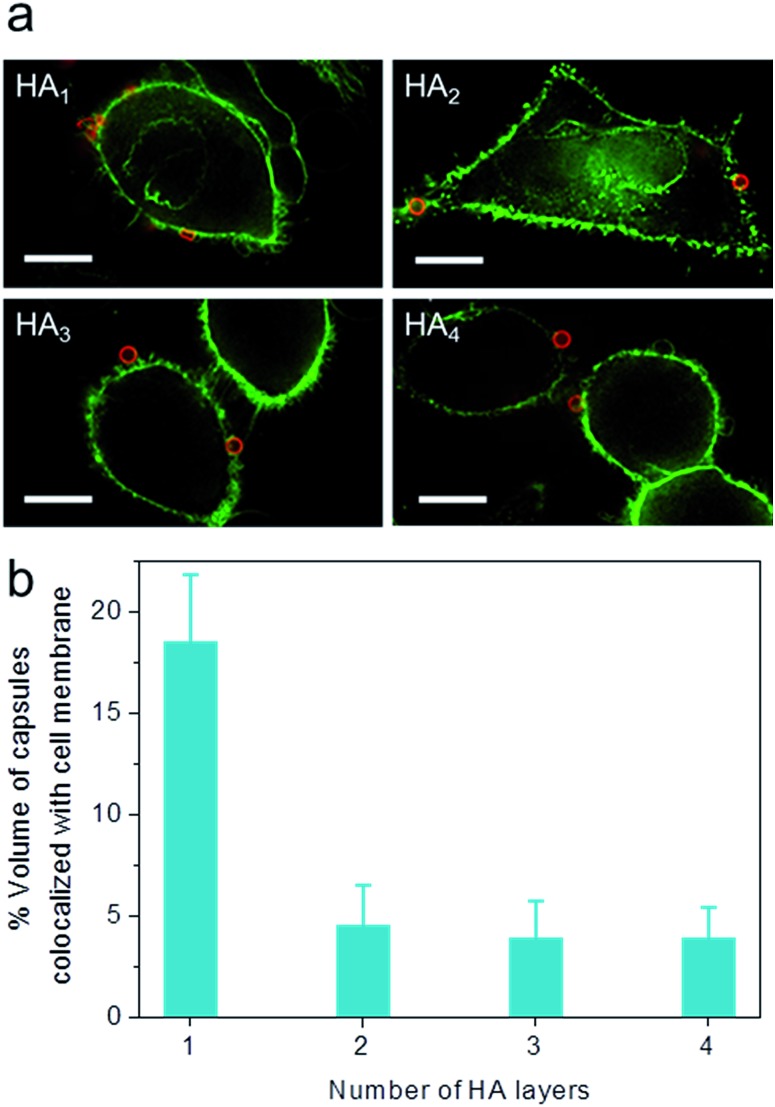
Cell surface binding of AF633-labeled HA capsules with different HA layers to HeLa cells, as determined by deconvolution microscopy. (a) Images of cells bound with capsules. (b) Percentage of capsule volume colocalized with cell membrane, analyzed *via* Imaris software. Cells were incubated with capsules (red) at a capsule to cell ratio of 100 : 1 for 2 h at 4 °C. Cell membranes were stained with AF488-WGA (green). Scale bars are 10 μm.

To elucidate the influence of capsule stiffness on cellular interactions, cell surface binding behavior of four types of HA capsules as a function of time was further studied by incubation at 4 °C. Consistent with the cellular association profile, the percentage of cells bound with the softest HA_1_ capsules (56–68%) was 2–3 times greater than that for the multilayered HA capsules during the 2 h incubation period (Fig. 4b). This is due to the fact that HA_1_ capsules with high flexibility are prone to deform upon contact with the cell membrane, thus leading to a higher contact area, as shown in the deconvolution microscopy images of cells bound with capsules (Fig. 5a). Furthermore, colocalization analysis of capsules with cell membranes (performed with the Imaris software package) indicated that the percentage of HA_1_ capsules colocalized with cell membranes was much higher than that for the multilayered capsules (19% *vs*. 4–5%) (Fig. 5b). The higher cell surface contact area as a result of deformation observed on HA_1_ capsules plausibly leads to higher cell membrane adhesion given the different capsules have similar surface chemistry with *ζ*-potentials ranging from –30 to –36 mV.

**Fig. 6:**
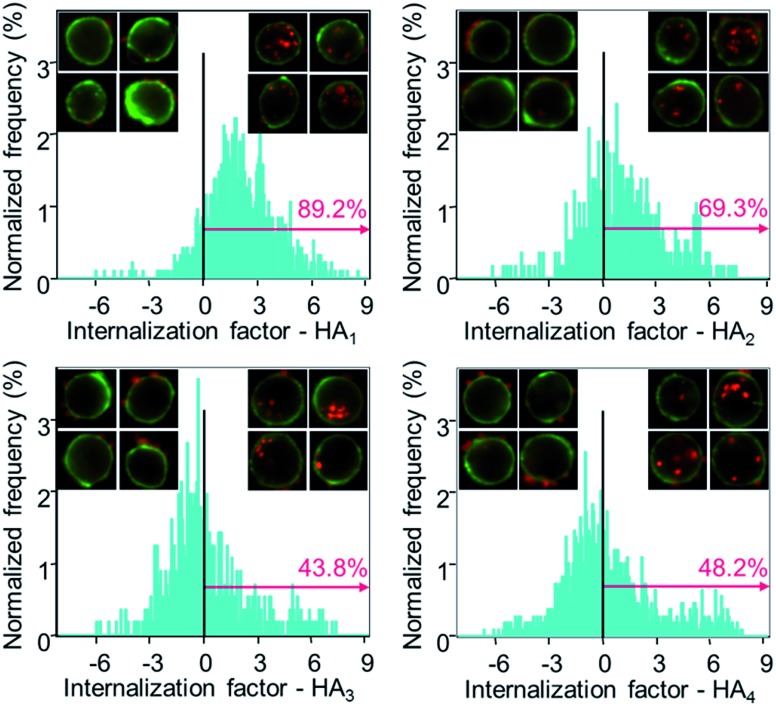
Quantification of the internalization of AF633-labeled HA capsules in HeLa cells *via* imaging flow cytometry. Cells were incubated with different HA capsules (HA_1_, HA_2_, HA_3_ and HA_4_) at a capsule to cell ratio of 100 : 1 for 24 h at 37 °C. Cell membranes were stained with AF488-WGA (green). The degree of internalization was expressed as the internalization factor (IF). Insets show representative images of cells with external surface-bound capsules (negative IF) and cells with internalized capsules (positive IF), respectively.

Subsequently, the cellular internalization tendency of various HA capsules was quantitatively analyzed *via* imaging flow cytometry, which integrates flow cytometry with fluorescence imaging and captures the bright field as well as fluorescence images of cells simultaneously, allowing quantitative and statistical analysis of the internalization from a large population of cells. Following incubation with HA capsules of varying stiffness for 24 h, cells were trypsinized and cell membranes were stained with Alexa Fluor 488-wheat germ agglutinin (AF488-WGA) to mark the outline of cells. Based on the acquired fluorescence images of cells and capsules, the intracellular or extracellular capsules can be quantitatively measured using the built-in internalization function in the IDEAS software to afford the internalization factor. The internalization factor is the ratio of the intensity inside the cell to the intensity of the whole cell, wherein a mask is created to define the inside of the cell.^40–42^ A positive factor relates to cells with mostly internalized capsules, whereas a negative factor refers to cells with mostly surface bound capsules. The results demonstrated that the percentage of cells with positive internalization factors decreased with increasing capsule stiffness, for which values of 89%, 69% and 44% were observed for HA_1_, HA_2_ and HA_3_ capsules, respectively (Fig. 6). In addition, it was found that the percentage of cells with internalized capsules decreased almost linearly with an increase in capsule stiffness from 7.5 to 27.2 mN m^–1^ (Fig. S5, ESI†), indicating a decrease in uptake with an increase in capsule stiffness. That is, the softer capsules underwent faster and more cellular internalization, which is consistent with a recent report that demonstrates softer PAH/PSS and DextS/PLArg capsules are transported faster into HeLa cells than stiffer capsules. Yi *et al.* performed a theoretical study on the cellular uptake of elastic nanoparticles based on the assumption that soft particles have changeable shapes while maintaining similar surface area and volume.^43^ They found that stiffer particles are more prone to achieve full membrane wrapping than the softer counterparts. However, an opposite trend was observed in our capsule system, which is likely due to the fact that hollow capsules are deformed and compressed during the cell uptake process, thereby resulting in decreased capsule volume and irreversible shape deformation,^24,44^ which would facilitate enveloping of capsules by the phagosomal cup. Therefore, softer capsules with higher flexibility can deform and alter their shape for phagosomal enveloping, thus promoting cellular uptake. Taken together, the cell surface binding, cellular association and internalization analysis validated that the capsules with higher stiffness were more prone to binding on the cell surface, rather than being internalized, possibly due to the difficulty in undergoing deformation.

**Fig. 7:**
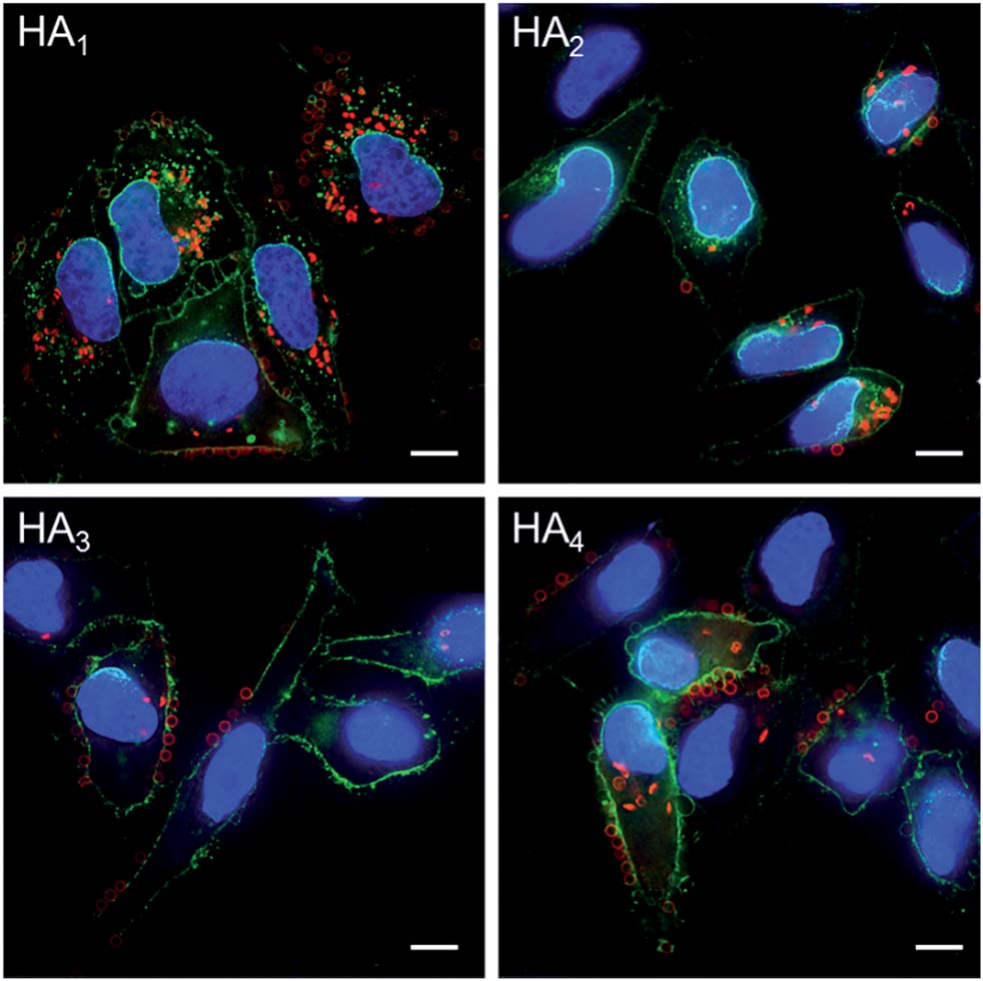
Cell internalization of AF633-labeled HA capsules with different stiffness in HeLa cells, as determined by deconvolution microscopy. Cells were incubated with capsules (red) at a capsule to cell ratio of 100 : 1 for 24 h at 37 °C. Cell membranes were stained with AF488-WGA (green) and nuclei were counterstained with Hoechst 33342 (blue). Scale bars are 10 μm.

The association and internalization behavior of HA capsules with varying stiffness was further corroborated by deconvolution microscopy. After 24 h incubation with HA capsules, cell membranes were stained with AF488-WGA and nuclei were stained with Hoechst 33342. Microscopy images showed that although most cells associated with capsules, the percentage of cells with internalized capsules decreased with increasing capsule stiffness (Fig. 7). Moreover, the internalization extent of the most flexible HA_1_ capsules was substantially higher than the stiffer capsules, most likely due to their ability to deform. It should be noted that all of the HA capsules lost their original spherical shape after internalization, regardless of their stiffness, which is in good agreement with previous reports that showed hollow polymeric capsules undergo deformation as a result of cell uptake.^24,44–46^ We next investigated the intracellular location of internalized capsules *via* the incubation of cells with AF633-labeled HA capsules for 24 h followed by immunostaining of lysosomes with the lysosome marker anti-LAMP1 antibody. The internalized HA capsules (red) are intensely colocalized with lysosomes (green) (Fig. S6, ESI†), thus affording yellow spots in the deconvolution microscopy images, despite the capsule stiffness. Overall, our data shows that stiffness plays an important role on the cellular interaction profiles of HA capsules, while the intracellular fate, including the capsule deformation and destination in lysosomes is consistently non-stiffness dependent.

## Experimental section

### Materials

Hyaluronic acid sodium salt (HA, *M*_w_ 47 kDa) was purchased from the Shandong Freda Biopharmaceutical Co., Ltd. (China). 2-Aminoethyl methacrylate hydrochloride (AEMA), 4-(4,6-dimethoxy-1,3,5-triazin-2-yl)-4-methylmorpholinium chloride (DMTMM), ethylene glycol dimethacrylate (EGDMA), *N*,*N*,*N*′,*N*′,*N*′′-pentamethyldiethylenetriamine (PMDETA, 99%), copper(ii) bromide (CuBr_2_, 99%), sodium ascorbate (NaAsc, ≥98%), *N*-(3-dimethylaminopropyl)-*N*′-ethylcarbodiimide (EDC), *N*-hydroxysuccinimide (NHS, 98%), hydrofluoric acid (HF), ammonium fluoride (NH_4_F), branched polyethyleneimine (PEI, *M*_w_ 25 kDa), sodium phosphate dibasic, sodium phosphate monobasic monohydrate and Dulbecco's phosphate-buffered saline (DPBS) were purchased from Sigma-Aldrich and used as received. Sodium acetate (NaOAc) was purchased from Merck. Functional monomers 2-(methacryloyloxy)ethyl trimethylammonium toluene sulfonate (METAOTs) and 2-(2-bromoisobutyryloxy)ethyl methacrylate (BIEM) were synthesized according to previously reported protocols^47,48^ but with some modifications and the detailed procedures are described in the ESI.† Alexa Fluor 633 (AF633) hydrazide reactive dyes, Alexa Fluor 488-Wheat Germ Agglutinin (AF488-WGA), Hoechst 33342 and Alexa Fluor 488 goat antimouse IgG were obtained from Invitrogen. 2,3-Bis(2-methoxy-4-nitro-5-sulfophenyl)-2*H*-tetrazolium-5-carboxanilide inner salt (XTT) was purchased from Promega. Mouse antihuman LAMP1 monoclonal antibody (clone H4A3) was purchased from BD Pharmingen. Nonporous SiO_2_ particles (50 mg mL^–1^, average diameter 2.4 ± 0.2 μm) were obtained from Microparticles GmbH (Berlin, Germany). The water used in all experiments was obtained from an inline Millipore RiOs/Origin system and had a resistivity greater than 18.2 MΩ cm.

### Characterization methods

Proton nuclear magnetic resonance (^1^H NMR) spectroscopy was conducted on a Varian Unity 400 MHz spectrometer using deuterated water (D_2_O) as the solvent and a sample concentration of approximately 4 mg mL^–1^. Differential interference contrast (DIC) and fluorescence microscopy images of HA particles and capsules were obtained using an inverted Olympus IX71 microscope equipped with a DIC slider (U-DICT, Olympus), a UF1032 fluorescence filter cube, and a 100× oil immersion objective (Olympus UPFL20/0.5NA, W.D1.6). Transmission electron microscopy (TEM) images were taken using a FEI Tecnai TF20 instrument with an operation voltage of 200 kV. Atomic force microscopy (AFM) experiments were performed with a JPK NanoWizard II BioAFM. Typical scans were performed in intermittent contact mode with MikroMasch silicon cantilevers (NSC/CSC). The film thickness of the capsules was analyzed using JPK SPM image processing software (version V.3.3.32).

### Force spectroscopy measurements and analysis

Mechanical characterization of the capsules was performed using a Nanowizard II AFM (JPK Instruments AG, Berlin, Germany) as described previously.^11,49^ The tipless cantilever (MLCT-O10, Bruker AFM Probes) was calibrated on a cleaned glass substrate to calculate the Inverse Optical Lever Sensitivity (InvOLS), and the spring constant was determined using the thermal tune method as described in literature.^50^ The resultant spring constant was evaluated as 0.07 N m^–1^. For the fabrication of the CP-modified cantilever, a spherical glass bead (7.5 μm radius, Polysciences Inc., USA) was attached to the tipless cantilever using an epoxy resin (Selleys Superstrength, Australia) *via* careful manual manipulation using AFM and associated optics, and left overnight. Prior to measurements, the cantilever with the attached glass bead was cleaned using oxygen plasma for 3 min.

For the force spectroscopy measurements, capsules were dispersed in water and allowed to settle onto a PEI-modified glass slide. The cantilever was then fully immersed in water and the InvOLS of the cantilever was measured again in water (23.9 nm V^–1^). Next, a force measurement was performed on a single immobilized capsule, which was visualized using an optical microscope (Leica DMI4000B). A piezo approach velocity of 500 nm s^–1^ was utilized for all measurements to avoid hydrodynamic effects. A force set-point (*i.e.*, maximal load) of 12 nN was utilized. Raw AFM voltage–displacement data were processed using JPK data processing software (v.4.4.28) to subtract the zero-force baseline, shift the data along the deformation axis to zero the initial contact point, subtract the effect of cantilever compliance, and extract data points. *γ* of the capsules was then evaluated as the linear gradient of the recorded force *vs.* deformation curves in the small deformation regime (50 nm). To achieve representative *γ* values for the capsules, at least ten different capsules were analyzed to generate the mean *γ*.

### Synthesis of macrocrosslinker and macroinitiator

Methacrylated hyaluronic acid (HA-AEMA) was synthesized through reaction of HA with AEMA in the presence of DMTMM (Scheme S1, ESI†). Briefly, to a 50 mL solution of HA (1.02 g, 2.49 mmol –COOH) in phosphate buffer (PB, 50 mM, pH 7.2) was added DMTMM (0.80 g, 2.89 mmol) and AEMA (0.25 g, 1.50 mmol). The reaction mixture was stirred for 3 days at room temperature. Then, the AEMA-modified HA was purified by dialysis against water, followed by lyophilization for 48 h. Yield: 92%, 0.96 g. AEMA functionality: 12 mol%. Fluorescently labeled HA-AEMA was obtained *via* reaction of HA-AEMA (50 mg) with AF633 hydrazide (50 μL, 1 mg mL^–1^ in DMSO) in the presence of EDC and NHS for 40 h, followed by dialysis and lyophilization.

The ATRP macroinitiator P(METAOTs-*co*-BIEM) was prepared by free radical copolymerization of METAOTs with BIEM (Scheme S2, ESI†). Briefly, METAOTs (1.37 g, 4 mmol), BIEM (1.12 g, 4 mmol) and AIBN (13.4 mg, 0.08 mmol) were dissolved in 7.2 mL of DMSO and degassed by argon bubbling. The solution was allowed to react at 100 °C for 2.5 h, and subsequently quenched by cooling in liquid N_2_ and exposure to air. The reaction mixture was diluted with 5 mL of methanol and precipitated into 150 mL of cold acetone/diethyl ether (15 : 1 v/v). Finally, the precipitated white powdery polymer was isolated by centrifugation and redissolved in water, followed by lyophilization. Yield: 42%, 1.04 g. BIEM percentage: 48 mol%.

### HA capsule formation

HA capsules were prepared in two steps. First, 200 μL of a negatively charged SiO_2_ particle suspension (50 mg mL^–1^, 2.4 μm) was centrifuged and washed with water (3 × 1 mL). The particles were then incubated in 1 mL of ATRP macroinitiator solution (1 mg mL^–1^) in NaOAc buffer (50 mM, pH 5.5) containing 0.5 M NaCl with constant shaking for 0.5 h at room temperature, isolated by centrifugation and washed with water (3 × 1 mL). Subsequently, the initiator-functionalized particles were dispersed in 600 μL of an aqueous stock solution containing HA-AEMA macrocrosslinker (17.0 mM AEMA), EGDMA crosslinker (1.7 mM), PMDETA (2.9 mM), CuBr_2_ (1.0 mM) and sodium ascorbate (19.3 mM). The mixture was agitated with an orbital shaker at room temperature for 2 h. Then, the particles were isolated by centrifugation, and washed with water (3 × 1 mL). This process represents the typical procedure for single HA layer formation and it was repeated multiple times to afford multilayered HA particles in so-called reinitiation and CAP_ATRP_ steps.

The aforementioned CAP_ATRP_-assembled HA particles were spun down and resuspended in 500 μL of PB (50 mM, pH 7.4), then 5 μL of AF633 hydrazide solution in DMSO (1 mg mL^–1^) and 500 μL of EDC solution in PB (10 mg mL^–1^) were added separately. The mixture was allowed to react in the dark for 24 h with constant shaking at room temperature. Afterwards, AF633-labeled HA particles were washed with PB (50 mM, pH 7.4, 3 × 1 mL), followed by water (3 × 1 mL), and finally redispersed in 100 μL of water.

HA capsules were obtained by mixing the particle suspension (100 μL in water) with 1.0 mL of ammonium fluoride (13.3 M) buffered hydrofluoric acid (HF) (5 M) at a volumetric ratio of 2 : 1 to remove the SiO_2_ template. [*Caution! HF solution is highly toxic. Extreme care should be taken when handling HF solution and only small quantities should be prepared.*] The capsules were subsequently centrifuged (3500*g*, 5 min) and washed thoroughly with PB (50 mM, pH 7.4, 3 × 1 mL).

### Stability of HA capsules

The stability test of AF633-labeled HA capsules was performed in DMEM medium supplemented with 10% FBS at 37 °C. Samples with a total capsule concentration of *ca.* 3 × 10^6^ capsule mL^–1^ were prepared by adding 4–6 μL of capsule suspension in PB (50 mM, pH 7.4) into 1.5 mL of DMEM medium. The total number of capsules in each sample over time was determined *via* flow cytometry. Experiments were performed in triplicate and data are presented as the mean ± standard deviation.

### Cell culture

HeLa cells were maintained in Dulbecco's modified Eagle's medium (DMEM, Gibco) with the addition of 10% FBS at 37 °C under a 5% CO_2_ humidified atmosphere and subcultured prior to confluence using trypsin.

### Cell viability analysis by XTT assay

The cytotoxicity of HA capsules toward HeLa cells was evaluated *via* XTT assays. In brief, HeLa cells were seeded into 96-well plates at a density of 5000 cells per well and incubated with different layered HA capsules at capsule to cell ratios of 1 : 1 to 200 : 1 for 48 h. After treatment, culture media were replaced by 50 μL of fresh DMEM media as well as 50 μL of XTT solution (5 mL of 1 mg mL^–1^ XTT in DPBS + 100 μL DMSO + 200 μL of 0.15 mg mL^–1^ PMS in DPBS) and the cells were incubated for a further 5 h. Then, the absorbance at a wavelength of 450 nm was measured using a Cary 50 Bio UV-Visible Spectrophotometer with a microplate reader. The relative cell viability (%) was determined by comparing the absorbance of untreated cells. Experiments were performed in sextuplicate and data are presented as the mean ± standard deviation.

### Cellular interaction analysis of capsules by flow cytometry

The percentage of cells associated or bound with HA capsules were assessed by flow cytometry. HeLa cells were seeded into 24-well plates at a density of 7.5 × 10^4^ cells per well and allowed to adhere overnight at 37 °C with 5% CO_2_. Then, AF633-labeled HA capsules were added at a capsule to cell ratio of 100 : 1 and incubated for predetermined time intervals. For cellular association studies, cells were incubated with capsules at 37 °C for 0.5, 1, 2, 4 and 8 h, respectively. At specified intervals, cells were washed three times with DPBS and harvested by trypsinization, followed by centrifugation at 300*g* for 5 min. The cell pellet was resuspended in DPBS and analyzed by flow cytometry (Apogee Flow). Cell surface binding experiments were performed similar to the cell association studies, except that cells were incubated with capsules at 4 °C for 0.5, 1 and 2 h, respectively. Cells were identified according to their scatter characteristics, and the percentage of cells bound with capsules was determined by acquisition of AF633 (FL5).

### Internalization analysis by imaging flow cytometry

Cells incubated with capsules were prepared as described previously. In brief, HeLa cells were seeded at 2 × 10^5^ cells per well into a 6-well plate and treated with AF633-labeled HA capsules at a capsule to cell ratio of 100 : 1 for 24 h. Following the treatment, cells were washed three times with DPBS, trypsinized, and collected by centrifugation at 300*g* for 5 min. Cell membranes were subsequently stained with AF488-WGA (0.2 μg mL^–1^) on ice for 20 min and cells were recovered *via* centrifugation at 4 °C. The cell pellet was resuspended in cold DPBS and kept on ice until analysis using imaging flow cytometry (Amnis Corporation, Seattle). Images of 4000 cells and their fluorescence intensity (FL6) arising from associated capsules were acquired using the 658/405 block filter. The cell internalization analysis was performed using the built-in internalization feature of IDEAS software on single focused cells associated with capsules.

### Cell imaging by fluorescence deconvolution microscopy

HeLa cells were plated at 3 × 10^4^ cells per well into 8-well Lab-Tek I chambered coverglass slides (Thermo Fisher Scientific, Rochester, NY, USA) and allowed to adhere overnight. Then, AF633-labeled HA capsules were added into the corresponding well to yield a final capsule to cell ratio of 100 : 1 and incubated for 2 h at 4 °C (binding) or 24 h at 37 °C. Subsequently, cells were washed three times with DPBS and fixed with 3% paraformaldehyde for 15 min at room temperature followed by washing three times with DPBS.

Cell membranes were stained *via* incubation with AF488-WGA (0.2 μg mL^–1^) on ice for 20 min, followed by washing with DPBS. Lysosomes were immunostained by permeabilizing cells with 0.1% Triton X-100 in DPBS for 5 min, incubating with mouse anti-human LAMP1 monoclonal antibody (2.5 μg mL^–1^) for 1 h, and detecting with a AF488-labeled goat anti-mouse IgG (2 μg mL^–1^) for 45 min at room temperature. Cell nuclei were counterstained using Hoechst 33342 (2.5 μg mL^–1^) for 15 min at room temperature. Following this, cells were washed three times with DPBS and immersed in 200 μL DPBS for microscopy observation. Fluorescence microscopy images were collected using a fluorescence deconvolution microscope (DeltaVision, Applied Precision) equipped with a 60× 1.42 NA oil objective and a standard DAPI/FITC/CY_5_ filter set. Images were processed with Imaris 6.3.1 (Bitplane) using the maximum intensity projection.

## Conclusions

This study provides insights into the effect of polymer capsule stiffness on cellular interactions. HA capsules with negligible shrinkage were prepared *via* the CAP_ATRP_ assembly of a HA-AEMA macrocrosslinker on SiO_2_ particles. Furthermore, the capsule wall thickness and associated capsule stiffness were finely controlled by changing the number of CAP_ATRP_ steps. These HA capsules revealed no obvious cytotoxicity toward HeLa cells. Using flow cytometry and imaging flow cytometry, the cellular binding, association, and internalization kinetics of HA capsules of varying stiffness were quantitatively determined. The results demonstrated that HA_1_ capsules with a *γ* of 7.5 mN m^–1^ possessed a much faster and higher cellular interaction with respect to binding, association and uptake. Furthermore, the internalization tendency of capsules significantly decreased with an increase in capsule stiffness, reflecting the slower and lower cellular uptake of stiffer capsules. Deconvolution microscopy images further confirmed the faster and greater cellular uptake of softer capsules. Although the stiffness plays an important role on the cellular uptake dynamics, deformation and lysosome localization were consistently the same for the different HA capsules when internalized. To further illustrate the stiffness-dependent cellular processing behavior, numerous cell lines and different capsules could be explored to investigate the important role of capsule stiffness on biological performance.
