# Supplementary material for: The role of capsule stiffness on cellular processing
Source: Chem Sci. 2015 Apr 29;6(6):3505–14. doi: 10.1039/c5sc00416k (PMC5492901; doi:10.1039/c5sc00416k)
Supplement: Supplementary file 1 [file SC-006-C5SC00416K-s001.pdf]

Electronic Supplementary Material (ESI) for Chemical Science.
This journal is © The Royal Society of Chemistry 2015

Electronic Supplementary Material (ESI) for Chemical Science.
This journal is © The Royal Society of Chemistry 2015

Supporting Information

The Role of Capsule Stiffness on Cellular Processing

Huanli Sun,[a] Edgar H. H. Wong,[b] Yan Yan,[a] Jiwei Cui,[a] Qiong Dai,[a] Junling Guo,[a] Greg G. Qiao,[*b] and Frank Caruso*[a]

a ARC Centre of Excellence in Convergent Bio-Nano Science and Technology, Department of Chemical and Biomolecular Engineering, The University of Melbourne, Parkville, Victoria 3010, Australia. Email: fcaruso@unimelb.edu.au

b Department of Chemical and Biomolecular Engineering, The University of Melbourne, Parkville, Victoria 3010, Australia. Email: gregghq@unimelb.edu.au

† Electronic Supplementary Information (ESI) is available. See DOI: 10.1039/b000000x/

Synthesis of METAOTs and BIEM

2-(Methacryloyloxy)ethyl trimethylammonium toluene sulfonate (METAOTs)

METAOTs was synthesized using a modified literature procedure.[1] Briefly, DMAEMA (11.0 g, 70 mmol) was dissolved in 100 mL of THF and cooled to 0 °C. Then, methyl *p*-toluenesulfonate (11.1 mL, 73.5 mmol) was added dropwise over 5 min. The quaternized monomer immediately began to precipitate, and the reaction mixture was stirred for an additional 3 h at room temperature. The precipitated solids were isolated by suction filtration, washed with THF (50 mL), redissolved in MeOH (30 mL), followed by precipitation into THF (150 mL). Finally, the monomer was collected by suction filtration and dried *in vacuo* yielding the title compound as a white powder (17.2 g, 72%). ^{1}H NMR (δ, ppm, d_4-MeOH): 7.71 (m, 2H, Ar-2,6***H***), 7.23 (m, 2H, Ar-3,5***H***), 6.16 (q, 1H, =C***H***), 5.71 (q, 1H, =C***H***), 4.61 (m, 2H, C(O)O-C$\boldsymbol{H}_2$), 3.76 (m, 2H, C$\boldsymbol{H}_2$N$^+$), 3.22 (s, 9H, (C$\boldsymbol{H}_3$)$_3$N$^+$), 2.36 (s, 3H, Ar-C$\boldsymbol{H}_3$), 1.96 (d, 3H, =CC$\boldsymbol{H}_3$).

2-(2-Bromoisobutyryloxy)ethyl methacrylate (BIEM)

BIEM was synthesized according to literature but with some modification.[2] In brief, HEMA (9.12 g, 70 mmol), DMAP (0.43 g, 3.5 mmol) and Et_3N (14.20 g, 140 mmol) were dissolved in 100 mL of dry diethyl ether (Et_2O) under an argon atmosphere and cooled to 0 °C. Then, a solution of bromo*iso*butylryl bromide (20.90 g, 91 mmol) in 10 mL of dry Et_2O was added dropwise over 30 min. The reaction mixture

was stirred at 0 °C for 1 h, and then at room temperature for a further 3 h. The reaction solution was subsequently filtered to remove precipitated ammonium salts and the filtrate was sequentially washed with 1 M HCl (2 × 50 mL), H_2O (2 × 50 mL), saturated $NaHCO_3$ (2 × 50 mL) and brine (2 × 50 mL). The organic phase was treated with decolorizing charcoal then dried over $MgSO_4$, filtered and concentrated at reduced pressure to yield a yellow crude product. The crude product was purified by vacuum distillation (72–76 °C, 8 mmHg) yielding the title compound as a colorless oil (14.7 g, 75%). ^{1}H NMR (δ, ppm, $CDCl_3$): 6.11 (s, 1H, =C***H***), 5.56 (s, 1H, =C***H***), 4.44 (m, 4H, C(O)O-C$\boldsymbol{H}_2$), 1.91 (s, 3H, =CC$\boldsymbol{H}_3$), 1.90 (s, 6H, C(Br)(C$\boldsymbol{H}_3$)$_2$).

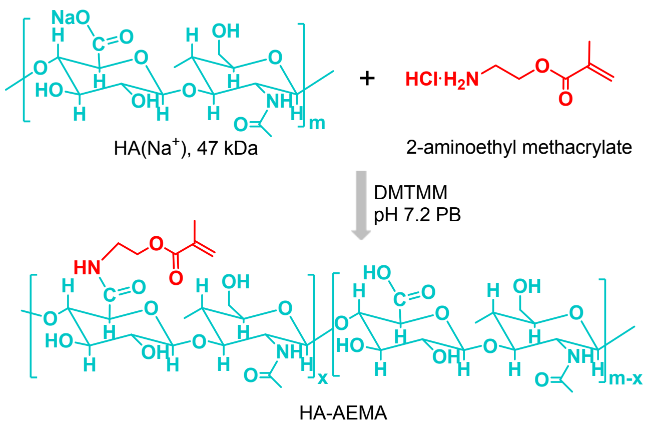

Scheme S1 Synthetic pathway for HA-AEMA macrocrosslinker.

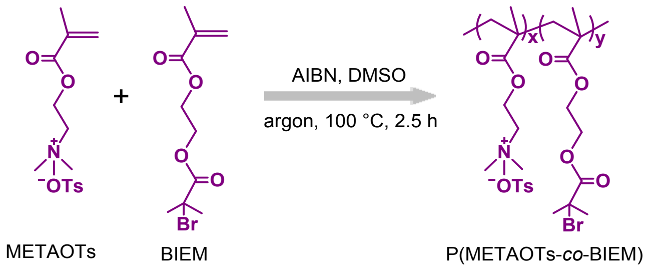

Scheme S2 Synthetic pathway for P(METAOTs-*co*-BIEM) ATRP macroinitiator.

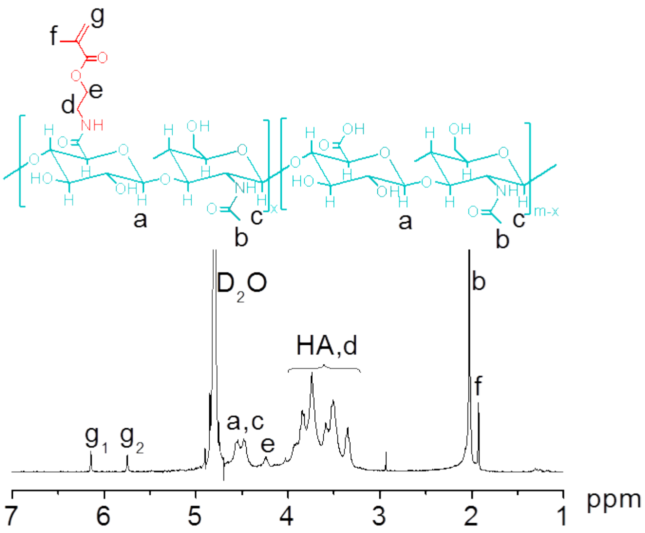

Fig. S1 ^{1}H NMR spectrum of HA-AEMA macrocrosslinker (400 MHz, D_2O).

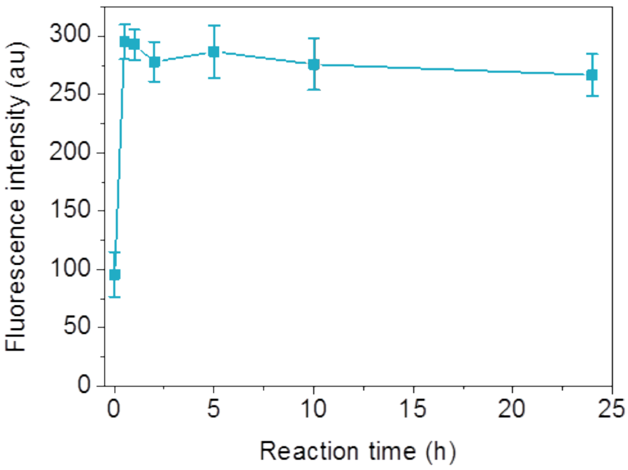

Fig. S2 Fluorescence intensity evolution of HA film growth on SiO_2 particles as a function of reaction time as followed by flow cytometry.

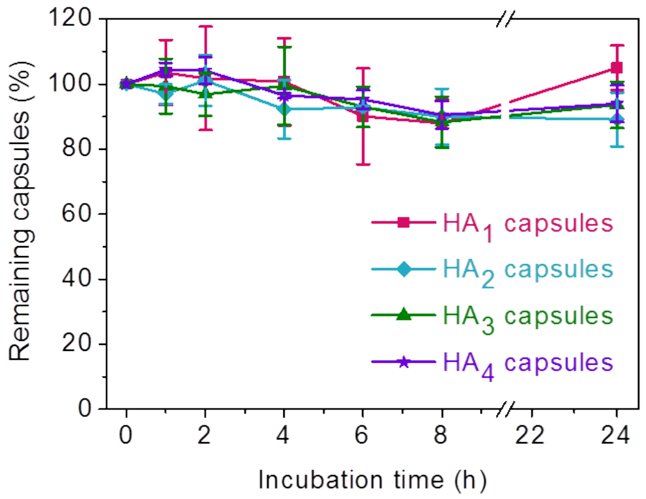

Fig. S3 The stability of AF633-labeled different-layered HA capsules in DMEM medium supplemented with 10% FBS at 37 °C, as determined *via* flow cytometry. Data are presented as the average ± standard deviation (n = 3).

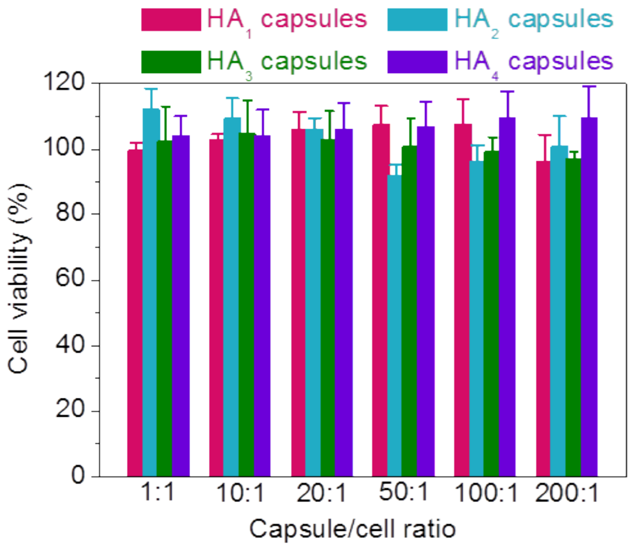

Fig. S4 Cell viability of HeLa cells after incubation with different layered HA capsules at varying capsule to cell ratios (1:1–200:1) for 48 h, as determined *via* XTT assay. The values were normalized to that of untreated cells, which were set at 100%. Data are presented as the average ± standard deviation (n = 6).

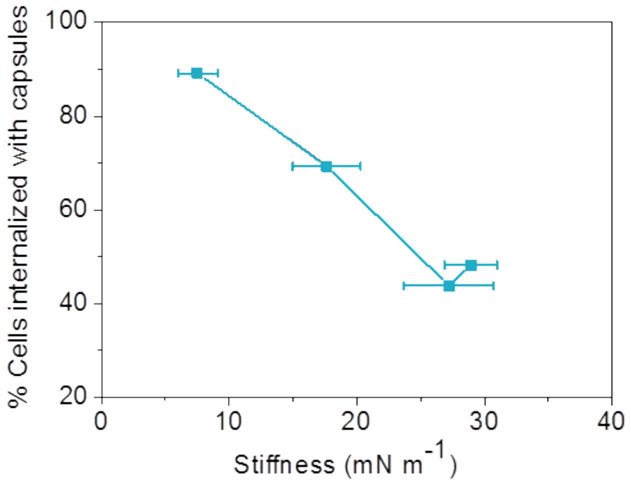

Fig. S5 Evolution of the percentage of cells internalized with HA capsules as a function of the stiffness (γ), as determined by imaging flow cytometry.

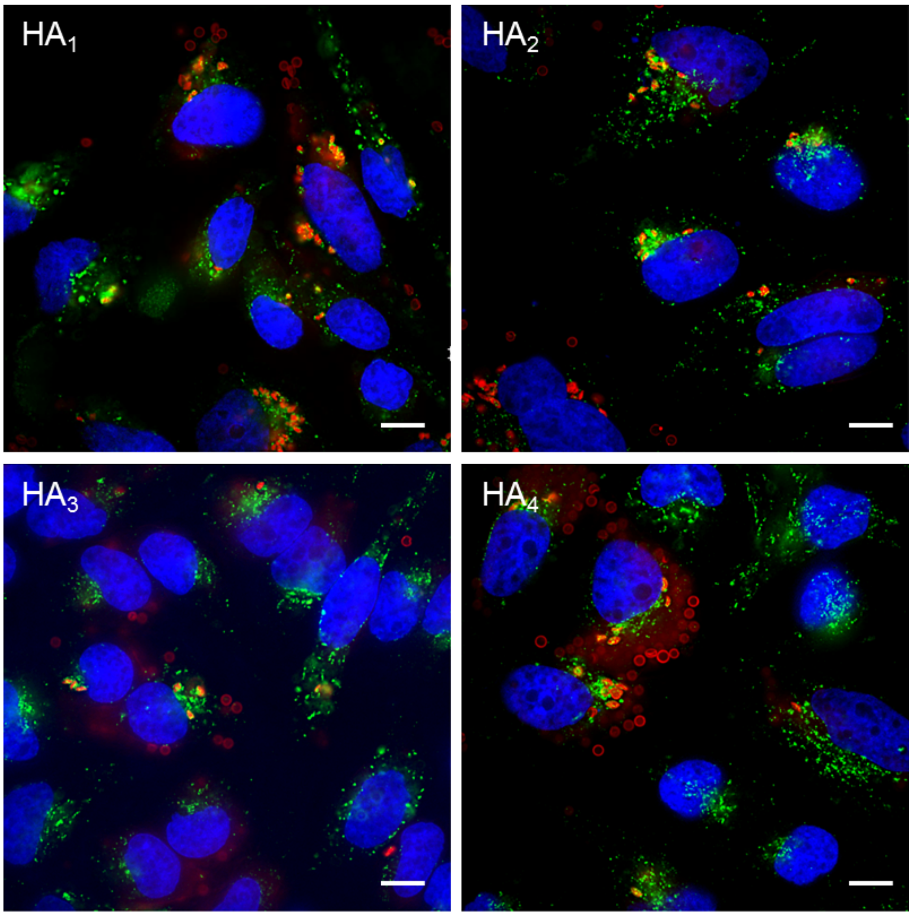

Fig. S6 Intracellular distribution of AF633-labeled HA capsules with different stiffness in HeLa cells as determined by deconvolution microscopy. Cells were incubated with capsules (red) at a capsule to cell ratio of 100:1 for 24 h at 37 °C. Lysosomes were immunostained with anti-LAMP1 antibody (green), and nuclei were counterstained with Hoechst 33342 (blue). Scale bars are 10 µm.

References

1 N. V. Tsarevsky, T. Pintauer and K. Matyjaszewski, *Macromolecules*, 2004, **37**, 9768-9778.

2 R. Venkatesh, L. Yajjou, C. E. Koning and B. Klumperman, *Macromol. Chem. Phys.*, 2004, **205**, 2161-2168.
